# Mass spectrometry protocol for tear fluid proteome profiling and biomarker discovery

**DOI:** 10.1016/j.xpro.2025.104262

**Published:** 2025-12-15

**Authors:** Kirstine Juul-Elbaek, Maria Peiris-Pagès, Vyacheslav Akimov, Irene Rebollido-Pedrido, Mark Reardon, Blagoy Blagoev, Petra Hamerlik

**Affiliations:** 1 Danish Cancer Institute, Copenhagen, Denmark; 2 Division of Cancer Sciences, University of Manchester, Manchester M20 4BX, UK; 3 Department of Biochemistry and Molecular Biology, University of Southern Denmark, Odense, Denmark; 4 Division of Medical Science, Chester Medical School, University of Chester, Chester, UK

**Keywords:** bioinformatics, cancer, proteomics

## Abstract

Here, we present a protocol for tear fluid proteomics using Schirmer strips and liquid chromatography-tandem mass spectrometry (LC-MS/MS). It includes tear sampling from human individuals and mice, followed by protein digestion, peptide desalting, and purification via solid-phase extraction. Supporting both data-dependent acquisition (DDA) and data-independent acquisition (DIA), this 5-h workflow maximizes protein recovery, minimizes contaminants, and enables reproducible biomarker discovery for ocular, neurodegenerative, and systemic diseases. Tear fluid’s non-invasive collection and rich proteome make it ideal for clinical proteomics and personalized medicine.

## Highlights

• Guidance for aseptic tear fluid collection using Schirmer strips in humans and mice
• Steps for protein extraction and digestion using GuaHCl, LysC, and trypsin
• Instructions for peptide desalting with SOLA plates for LC-MS/MS analysis
• Procedures for minimizing variability in tear-based biomarker discovery

## Abstract (editor highlights)

Publisher’s note: Undertaking any experimental protocol requires adherence to local institutional guidelines for laboratory safety and ethics.

## Graphical abstract

**Figure:**
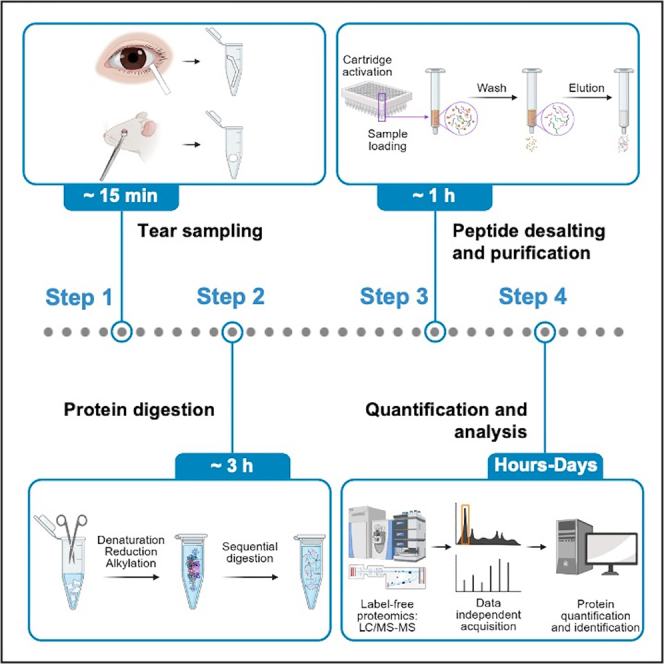

## Before you begin

Proteins represent essential targets in the search for biomarkers, as they can signal healthy or diseased states, responses to pharmacological interventions, and other relevant clinical information. To date, over 15,000 different proteins have been identified in major human body fluids.^1^ The most commonly studied plasma proteome exhibits an exceptionally broad dynamic range, spanning up to 10–12 orders of magnitude, primarily due to the presence of highly abundant proteins such as albumin and immunoglobulins. This complexity necessitates depletion strategies to enable the detection of low-abundance biomarkers. Tear fluid proteomics is gaining attention for biomarker discovery due to its non-invasive collection, and despite their small volume (3-10 μL), rich proteome, with a protein concentration ranging from 6 to 11 mg/mL.^2^^,^^3^^,^^4^^,^^5^ In healthy individuals, human tears contain >1,000 proteins.^5^ Unlike the collection of plasma or cerebrospinal fluid, tear fluid collection is minimally invasive, making tears an ideal source for biomarker discovery for diagnostic, prognostic and monitoring purposes.^4^ This study presents an optimized protocol for the extraction, digestion and preparation of tear fluid proteins, with a focus on maximizing protein recovery, minimizing contaminants, and achieving higher reproducibility, all of which are critical for clinical proteomics. Our protocol details the isolation and processing of tear fluid collected using the Schirmer strip test for Liquid Chromatography-Tandem Mass Spectrometry (LC-MS/MS) analysis.^6^ Before starting, define research goals and control factors affecting protein stability (e.g., temperature). Prepare all reagents and equipment in advance. This 5-h protocol improves tear proteome reliability, supporting clinical proteomics, biomarker discovery, and personalized medicine.

### Innovation

This protocol presents a robust and reproducible workflow for high-throughput proteomic profiling of tear fluid using Schirmer strips and LC-MS/MS. It introduces several key innovations that advance tear fluid analysis for biomarker discovery. First, the protocol optimizes protein extraction and digestion from low-volume tear samples, achieving high peptide yields (∼105 μg per eye) with minimal variability. Second, it incorporates a novel tube-puncture method for efficient peptide recovery and offers an alternative in-strip digestion strategy for challenging samples, enhancing flexibility across experimental designs. The workflow integrates solid-phase extraction using SOLA plates to improve peptide purity and reduce background noise and supports both Data Dependent (DDA) and Data Independent Acquisition (DIA) modes for comprehensive proteome coverage. Importantly, the protocol demonstrates that 1-min tear collection yields comparable proteomic depth to 5-min sampling, enabling shorter, patient-friendly collection times without compromising data quality. This method is compatible with both human and murine tear samples and is designed for scalability and clinical translation. By combining non-invasive sampling, optimized protein processing, and reproducible quantification, this protocol significantly enhances the utility of tear fluid in clinical proteomics and personalized medicine.

### Institutional permissions (if applicable)

All procedures using human study participants were conducted in compliance with institutional guidelines and approved by the University of Manchester Research Ethics Committee (UREC 2024-20051-36638). All animal work was carried out under a UK Home license (PP6556294) and approved by the University of Manchester and the CRUK Manchester Institute review committees.

### Tear sampling

**Timing: ∼10–15 min (for step 1)**

**Timing: ∼5 min (for step 2)**

**Timing: ∼2 h (for step 3)**

The Schirmer tear test is a widely used clinical method for assessing tear fluid production in conditions such as dry eye disease or Sjögren’s syndrome. This procedure involves placing a Whatman filter paper strip into the lower conjunctival fornix for five minutes, allowing it to absorb tear fluid effectively. The test captures both intracellular and extracellular proteins, along with other molecules such as DNA, lipids, and metabolites from the ocular surface.^7^ Schirmer strips are often preferred over other methods, such as capillary tubes, due to their superior efficiency and safety in collecting tear fluid. For this study, tear fluid samples were collected using Schirmer strips.

This method is specifically designed to assess the proteome profile of tear fluid using label-free proteomics. It is essential to collect samples from individuals who are not wearing cosmetics/makeup at the time of sampling (refer to problem 1 in the troubleshooting section). After collecting the samples, the strips should be placed into 1.5 mL Protein Low Binding Microcentrifuge Tubes (LoBind) that have been pre-cooled on dry ice and then stored at −80°C until further use (see problem 2 in the troubleshooting section).

**Figure 1:**
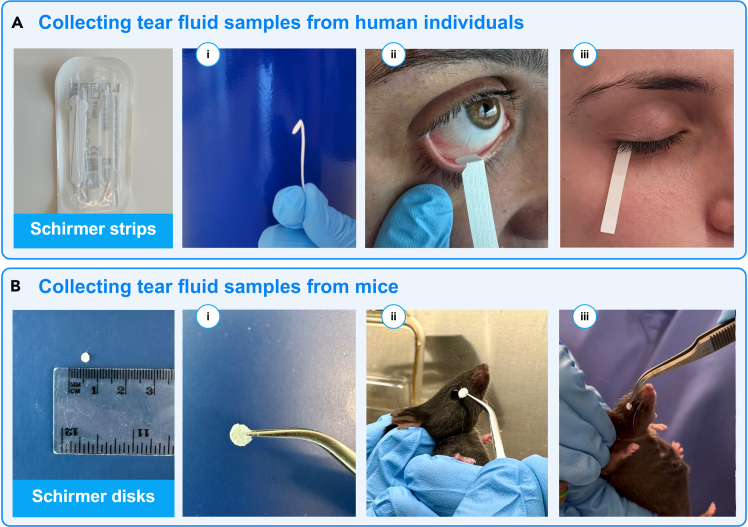
Collection of tear fluid (A) Human subjects: i) Schirmer strips are bent while still inside their sterile packaging; ii) Schirmer strips are placed in the lower eyelid of human subjects for tear collection; iii) Tear fluid is absorbed by capillary action into the strip. (B) Mice: i) 3 mm diameter disks are prepared from Schirmer strips using a paper punch; ii) Tears are collected by inserting a disk into the lower eyelid of the mouse for 15 seconds per eye using sterile tweezers; iii) Tear fluid is absorbed by capillary action into the disks. Created in BioRender. Hamerlik, P. (2025) https://BioRender.com/5czcwg7.

1. Tear Fluid Collection using the Schirmer tear test from human individuals (Figure 1A).
  a. Sample tear fluid from the left and right eyes of individuals using a standard Schirmer’s tear test (SKU 2701026; Haag-Streit UK Ltd, United Kingdom) without the use of anesthetics.
    i. Prepare the Schirmer’s strips by bending them at the notch while still in the sterile packing.
    ii. Instruct the subject to loop upward.
    iii. Carefully insert the bent end of a strip into the space between the left lower eyelid and eye (inferior fornix, positioned near the palpebral margin).
    iv. Instruct the subject to gently close their eyes, refraining from squinting.
    v. After a 5-min, carefully remove and immediately transfer the strip to a pre-cooled Protein LoBind Eppendorf tube (EP0030010811; Eppendorf, Germany) placed on dry ice.
    vi. Repeat the procedure for the right eye.
    vii. Store strips at −80°C until further processing.

***Note:*** The entire strip will be used for downstream analysis. If the subject’s Schirmer strip shows less than 5 mm of wetting after 5 min, the protein yield may be reduced, and thus the sample is ineligible for downstream analysis.

**CRITICAL:** To prevent contamination, collection must be performed using aseptic techniques, including the use of sterile gloves.

2. Collecting tear fluid samples using the Schirmer tear test from mice (Figure 1B).
  a. Sample tear fluid from mice using disks (3 mm diameter) generated by punching standard Schirmer’s tear test strips (SKU 2701026; Haag-Streit UK Ltd, United Kingdom).
    i. Use sterile forceps to hold the Schirmer strip and align the 3 mm puncher straight over it.
    ii. Press down firmly to cut out a disk, then move the strip slightly and repeat the punching process to create additional disks.
    iii. Once finished, release and carefully extract the disks from the puncher. Typically, each Schirmer strip yields approximately 10 disks.
    iv. Before use, sterilize the strips under UV light of a laminar airflow cabinet for 20 min to ensure aseptic conditions.
    v. Lift the mouse from its home cage to a wire lid, or any other surface that provides traction to the mouse.
    vi. Using your thumb and index finger, scruff the loose skin over the neck, shoulders and back.
    vii. Use the other hand, holding tweezers, insert the disk into the space between the left lower eyelid and eyeball.
    viii. Following a 15-s absorption period, transfer the disk to pre-cooled Protein LoBind Eppendorf tubes (Eppendorf, Germany) on dry ice.
    ix. Repeat the procedure for the right eye and store at −80°C until use.

***Note:*** No anesthetics or pharmacological stimulators of lacrimal glands are required for this procedure. The mouse’s head should be immobilized, and the front arms slightly splayed. Lift the mouse with the hand performing the scruff hold. Secure the tail between the ring or small finger and the palm of the same hand.

***Note:*** If immediate protein digestion is feasible post-collection, sample freezing is unnecessary. Otherwise, tear fluid is stored at <−80°C until processing.

**CRITICAL:** Gloves need to be worn to avoid contamination with skin cells.

**CRITICAL:** Optimal protein yield relies on maximizing the speed of tear fluid freezing (see problem 2 in troubleshooting).

3. Preparation of reagents and equipment for sample processing.
  a. Preheat Thermomixer or block heater to 37°C.
  b. Prepare all necessary buffers in advance and ensure they reach room temperature before tear processing.

**Figure 2:**
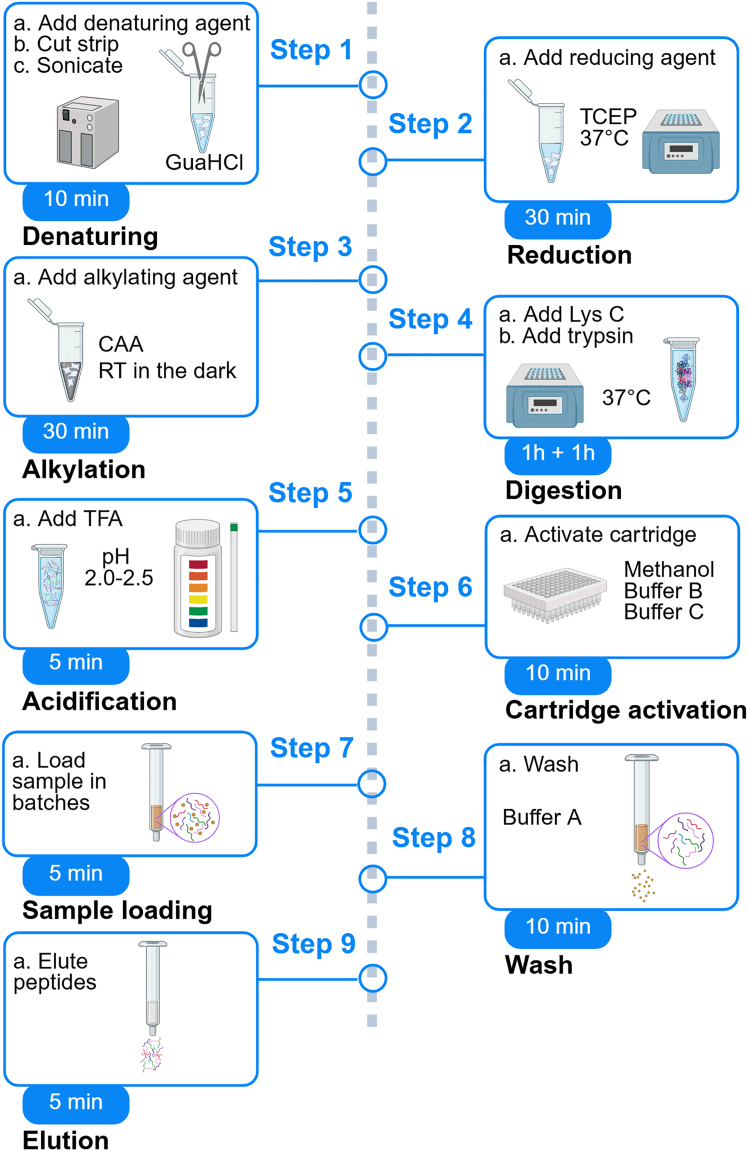
Schematic of an Experimental workflow Step-by-step method for high-throughput tear fluid sample preparation for proteomic profiling. Created in BioRender. Hamerlik, P. (2025) https://BioRender.com/e04zalo.

Before you begin, carefully review schematic shown in Figure 2. It highlights individual protocol steps as proteins undergo denaturation using a chaotropic agent such as GuaHCl and sonication, reduction with TCEP, and alkylation with CAA to irreversibly prevent the free sulfhydryl on cysteine residues from reforming disulfide bonds. The denatured, reduced, and alkylated proteins are then enzymatically digested by endoproteinases (Lys C and trypsin) to hydrolytically break down peptide bonds and fragment proteins into peptides. Finally, desalting is performed to remove salt and buffers to prevent background noise using SOLA Solid-Phase Extraction (SPE) cartridges (Thermo Scientific 60309-001), and peptides are eluted in MS-compatible buffers. These peptides are subsequently separated and analyzed by LC-MS/MS.

## Key resources table

**Table:** 

REAGENT or RESOURCE	SOURCE	IDENTIFIER
**Biological samples**		

Human Tears	Female healthy volunteer	N/A
Mouse Tears	Female mouse, 6 weeks old	C57BL/6J

**Chemicals, peptides, and recombinant proteins**		

Ammonium bicarbonate (ABC)	Sigma	Cat# 5330050050
Guanidine hydrochloride (GuaHCl)	Sigma	Cat# 50933
2-Chloroacetamide (CAA)	Sigma	Cat# C0267
Tris(2-carboxyethyl) phosphine hydrochloride solution (TCEP)	Sigma	Cat# 646547
Trifluoroacetic acid, Optima LC/MS grade (TFA)	Fischer Scientific	Cat# 10723857
Lysyl endopeptidase, LC/MS grade (Lys C)	Fujifilm Wako	Cat# 125-05061
Sequencing LC/MS grade Modified Trypsin	Promega	Cat# V511B
Acetonitrile, LC-MS Grade CHROMASOLV, tested for UHPLC-MS, 99.9 % (ACN)	Honeywell	Cat# 15601400
LC-MS Grade water	Sigma	Cat# 1153331000
Methanol, Optima LC/MS grade	Fischer Scientific	Cat# 10767665
Water with 0.1% (v/v) formic acid (**Buffer A**)	Sigma	Cat# 1590132500
80% Acetonitrile, 20% Water with 0.1% Formic Acid, Optima LC/MS grade (**Buffer B**)	Fischer Scientific	Cat# LS122-500

**Software and algorithms**		

Spectronaut or alternative software	Biognosys	Version 18.4.231011.55695

**Other**		

Protein Low-binding (LoBind) tubes	Eppendorf	Cat# 30108116
Protein Low-binding (LoBind) PCR plate	Eppendorf	Cat# 737-0186
Iris scissors single-use	Teqler	Cat# T710102
22G needle (or 23G)	BD	Cat# 301000
pH-Box pH-indicator paper pH 0.5–13.0	Merck	Cat# 1095650001
MicroAmp Optical Adhesive Film	Thermo Fisher scientific	Cat# 4311971
Deepwell plate 96/1000 for SOLA plate	Eppendorf	Cat# 30504216
SOLA™ Solid Phase Extraction (SPE) Plates	Thermo Fisher scientific	Cat# 60209-001
Pipette Tip 50-1000 μL Racked Low Retention pk768	Mettler Toledo Ltd.	Cat# 30389167
Borosilicate glass bottles, 100 mL	Duran	Cat# B2B9268
Thermomixer F1.5	Eppendorf	Cat# EP5384000012
Schirmer tear test	Haag-Streit	Cat# 4701001
ScoutPro scales	Ohaus	Model SP202
Benchtop centrifuge with adaptor for plates	Eppendorf	Model 5810 R
Ultrasonic bath	Grant	Model XB3
Laboratory Chemical fume cupboard	S+B	N/A
Nanodrop (optional)	IMPLEN GmbH	NanoPhotometer N60
Centrifuge concentrator (optional)	Eppendorf	Concentrator plus

## Materials and equipment

***Alternatives:*** The use of specific brands of equipment (such as a centrifuge from Eppendorf) is not essential; any equipment capable of meeting the necessary temperature and specification requirements (such as securely accommodating 1.5 ml tubes or plates) can serve the purpose adequately. Similarly, alternative chemical fume hoods, scales or sonicators with similar specifications can be utilized instead of those listed in the key resources table.

***Alternatives:*** Using chemicals from manufacturers other than those specified in the key resources table is allowed, provided they are of LC/MS Grade.

**CRITICAL:** Prepare all buffers in a fume hood using autoclaved glassware that has not been cleaned with detergent. Bottles can be reused for preparing the same solvent or buffer.

**Table:** ABC buffer

Reagent	Final concentration	Amount
Ammonium bicarbonate	25 mM	198 mg
LC/MS grade water	N/A	Up to 100 mL

**CAUTION:** Ammonium bicarbonate is a skin and eye irritant. Appropriate PPE (e.g., gloves, goggles and a laboratory coat) should be used while working with this substance.

**Table:** GuaHCl solution

Reagent	Final concentration	Amount
GuaHCl	8 M	7.64 g
ABC	25 mM	Up to 10 mL

**CAUTION:** GuaHCl is a skin and eye irritant. Appropriate PPE (e.g., gloves, goggles and a laboratory coat) should be used while working with this substance.

**Table:** CAA

Reagent	Final concentration	Amount
CAA	0.5 M	468 mg
LC/MS grade water	N/A	Up to 10 mL

**CAUTION:** CAA is toxic upon ingestion, may cause an allergic skin reaction, and a suspected reproductive toxin. Appropriate PPE (e.g., gloves, goggles and a laboratory coat) should be used while working with this substance.

**Table:** 20 % TFA

Reagent	Final concentration	Amount
TFA	20%	2 mL
LC/MS grade water	N/A	8 mL

**Table:** 10 % TFA

Reagent	Final concentration	Amount
TFA	10%	1 mL
LC/MS grade water	N/A	9 mL

***Note:*** To prepare a 10% solution, you can also mix 1 mL of TFA (20%) with 1 mL of LC/MS grade water.

**CAUTION:** TFA is an irritant and corrosive to skin, eyes and respiratory tract tissues, acutely toxic by inhalation and ingestion, and corrosive to metals. Appropriate PPE (E.g. gloves, goggles and a laboratory coat) should be used while working with this substance. A chemical fume hood should be used when working with TFA.

**Table:** Lys C solution

Reagent	Final concentration	Amount
Lysyl endopeptidase	0.25 μg/μL	20 ug
ABC	25 mM	80 μL

**Table:** Trypsin

Reagent	Final concentration	Amount
Trypsin	0.25 μg/μL	100 ug
ABC	25 mM	400 μL

**CRITICAL:** Do not vortex Lys C and trypsin when resuspending or thawing. Only gentle pipetting or inversion.

**Table:** Buffer C (3% ACN, 1% TFA)

Reagent	Final concentration	Amount
ACN	3%	3 mL
20% TFA	1%	5 mL
LC/MS grade water	N/A	92 mL

**Table:** Buffer D (40% ACN, 0.1% FA)

Reagent	Final concentration	Amount
Buffer A	0.1%	50 mL
Buffer B	80% ACN, 0.1% FA	50 mL

**CAUTION:** Acetonitrile (ACN) is highly flammable and considered an acutely toxic solvent upon skin exposure. Formic acid is also flammable, irritant, corrosive to skin, eyes, respiratory tract, acutely toxic by ingestion or inhalation, and corrosive to metals. Appropriate PPE (e.g., gloves, goggles, a laboratory coat and a chemical fume hood) should be used while working with these substances.

## Step-by-step method details

### Protein digestion

**Timing: ∼4 h**

This section outlines the procedures for preparing peptides for mass spectrometry analysis. These steps were refined to maximize yields when isolating tear proteins absorbed in Schirmer tear strips.

**Figure 3:**
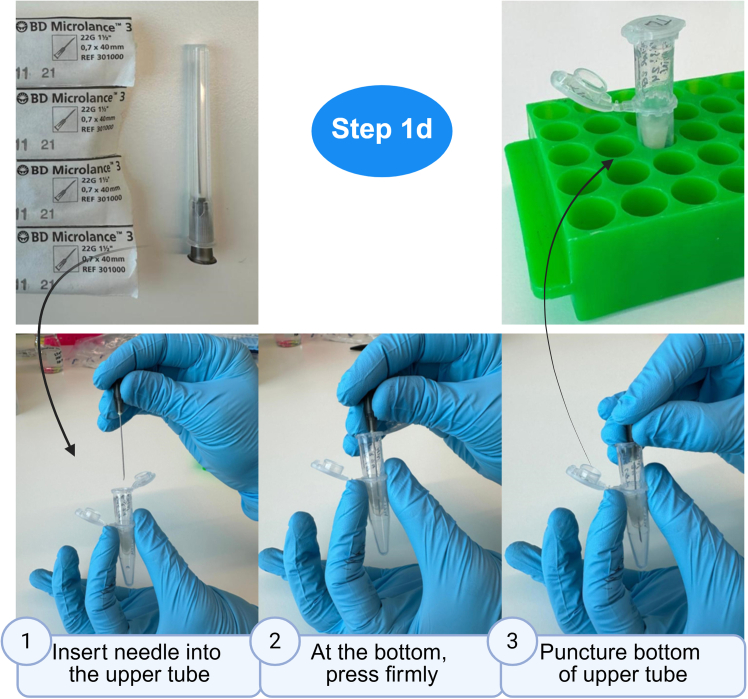
Puncture of tubes Insert a 22G needle into the upper tube until it reaches the bottom. Press firmly to puncture the bottom of the upper tube. Remove the needle; the tubes are now ready for use. Created in BioRender. Hamerlik, P. (2025) https://BioRender.com/tm7hc67.

1. Denaturation.
  a. Prepare a fresh solution of 8 M GuaHCl in 25 mM ABC buffer.
  b. Remove the tear samples from the freezer and place them on dry ice.
  c. Cut the strips inside the LoBind tube with single-use scissors into smaller pieces (ideally less than 5 mm × 5 mm).
  d. Transfer the samples to laboratory temperature (18°C–24°C) and immediately add 187.5 μL of GuaHCl solution per tear strip/LoBind tube.
    ***Note:*** Store ABC buffer at standard laboratory temperature 18°C–24°C for a period of 3 months.
    ***Note:*** Guanidine hydrochloride is used as it outperforms other denaturing agents (higher denaturing strength, stability under harsh conditions, and chaotropic properties that enhance protein solubility and extraction efficiency).
    **CRITICAL:** Ensure that all strip fragments are fully submerged in the liquid before proceeding to the subsequent sonication step 2. Spin down if needed.
  e. Sonicate for 5 min in an ultrasonic bath (∼50–60 Hz).
  f. Label new Protein LoBind tubes accordingly. Use a needle to puncture the bottoms of the previous tubes and stack them on top of the newly labelled tubes (Figure 3).
  g. Spin at 16,000 × *g* for 10 min at laboratory temperature (18°C–24°C). Remove and discard the top tubes containing the strips.
    **CRITICAL:** Ensure that Eppendorf tube lids face outward in the centrifuge to prevent them from being damaged during spinning.
    ***Alternatives***: For in-strip digestion, proceed to step 2 after step 1c (see problem 3 in troubleshooting).
2. Reduction.
  a. Reduce by adding to the volume collected from the previous step 3.75 μL of 0.5 M TCEP (final concentration of TCEP will be 10 mM). Centrifugation in the previous step should result in minimal liquid loss (<10%).
  b. Incubate at 37°C for 30 min in a thermomixer, a heat block or similar.

***Note:*** Prepare TCEP fresh before use.

3. Alkylation.
  a. Alkylate by adding 4.1 μL of 0.5 M CAA (final concentration of CAA will be 11 mM).
  b. Incubate for 30 min at laboratory temperature (18°C–24°C) **in the dark.**
  c. Stop reaction by adding 562.5 μL of 25 mM ABC to achieve a final concentration of 2 M GuaHCl.

***Note:*** Store 0.5 M CAA at - −80°C in 1 ml aliquots in opaque tubes (CAA is photosensitive) for a period of 3 months maximum.

***Note:*** Prepare 11 mM CAA fresh before use.

4. Sequential digestion.
  a. Digest by adding 6 μL of Lys C solution for 1 h at room temperature (1.5 μg of LysC per sample - a minimum of 1 μg is recommended).
  b. Dilute Lys C by adding 750 μL of 25 mM ABC to achieve a final concentration of 1 M GuaHCl.
  c. Digest by adding 6 μL of trypsin solution (1.5 μg of trypsin per sample - a minimum of 1 μg is recommended). Place the tubes into the Thermomixer or heat block at 37° C for 1–1.5 h.

***Alternatives:*** Leave the tubes **overnight** at laboratory temperature (18°C–24°C) (see problem 4 in troubleshooting).

***Note:*** Store Lys C solution at −80°C in aliquots for a period of 3 months maximum.

***Note:*** Do not vortex after the addition of Lys C or trypsin. Gently mix by inverting the tube. Trypsin digestion cleaves proteins specifically at the carboxy-terminal (C-terminal) of Arg and Lys residues. However, exceptions to this cleavage pattern have been observed empirically. Pre-digestion with another protease, Lys C, helps improve digestion efficiency and minimizes missed cleavages, particularly at lysine residues preceding prolines.^8^

5. Sample acidification.
  Acidify samples at laboratory temperature (18°C–24°C) to stop enzymatic activity.
  a. Add 20–30 μL of 10% TFA.
  b. Vortex for 5 s.
  c. Confirm that the pH of the samples has decreased to 2.0–2.5 by pipetting ∼2 μL of the acidified sample mixture on a pH indicator strip (see problem 5 in troubleshooting).
    ***Note:*** Store 10% TFA at standard laboratory temperature 18°C–24°C for a period of 3 months.

### Protein desalting and purification via solid-phase extraction

**Timing: ∼1 h**

This section outlines the solid-phase extraction (SPE) technique, where samples are loaded onto a solid-phase sorbent contained within a cartridge (in this case, the SOLAμ plate). The analytes of interest selectively bind to the sorbent while interfering compounds are washed away. Elution with an appropriate solvent releases the analytes, yielding a purified extract suitable for downstream analysis.

**Figure 4:**
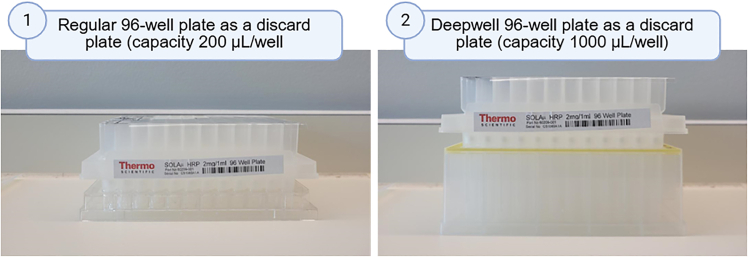
Centrifugation process using either regular 96-well plates (left) or high-capacity Deepwell discard plates (right), depending on centrifuge capacity Created in BioRender. Hamerlik, P. (2025) https://BioRender.com/doaxc5h.

6. Cartridge activation.
  a. Place the SOLA plate in a 96-well waste plate (Figure 4).
  b. Add 200 μL of LC/MS-grade methanol.
  c. Centrifuge at 450 × *g* for 1 min at room temperature and discard the flow-through.
  d. Add 200 μL of Buffer B.
  e. Centrifuge at 450 × *g* for 1 min at room temperature and discard the flow-through.
  f. Wash with 200 μL of Buffer C.
  g. Centrifuge at 450 × *g* for 1 min at room temperature and discard the flow-through.
  h. Repeat wash (steps 6f and 6g).

***Note:*** When handling fewer than 96 samples, only activate and utilize the necessary wells.

***Note:*** Do not allow the filters on the SOLA cartridge to dry out.

***Note:*** Store Buffer C at standard laboratory temperature 18°C–24°C for a period of 3 months.

**CAUTION:** Methanol is highly flammable, a suspected fetal toxin, an eye irritant, and considered an acutely and chronically toxic solvent. Appropriate PPE (E.g. gloves, goggles, a laboratory coat and a chemical fume hood) should be used while working with this substance.

7. Load acidified samples and wash.
  a. Transfer digested peptides using low-retention tips to the 96-well SOLA plate in batches of 200–1,000 μL, depending on the type of discard plate used (Figure 4).
    ***Note:*** If your centrifuge can accommodate larger vessels, utilize discard plates with higher volume capacity. This modification allows for increased sample processing and potentially reduces the number of centrifugation cycles required (Figure 4).
    ***Alternatives:*** Load half of the sample and store the remaining half at −80°C for future analysis, if needed.
  b. Centrifuge at 450 × *g* for 1 min at laboratory temperature (18°C–24°C) and discard the flow-through each time.
8. Wash.
  a. Wash with 200 μL of Buffer A.
  b. Centrifuge at 450 × *g* for 1 min at laboratory temperature (18°C–24°C) and discard the flow-through.
  c. Repeat wash (steps 8a and 8b).
9. Elution of peptides.
  a. Place the SOLA plate containing washed peptides in a clean collection LoBind 96-well PCR plate.
  b. Elute peptides by adding 30 μL of Buffer D.
  c. Centrifuge at 450 × *g* for 1 min at laboratory temperature (18°C–24°C).
  d. Repeat the elution step by adding 30 μL of Buffer D.
  e. Centrifuge at 450 × *g* for 1 min at laboratory temperature (18°C–24°C). The final volume of the sample will be 60 μL at this point.

***Note:*** Store Buffer D at standard laboratory temperature 18°C–24°C for a period of 3 months.

10. Storage of samples for LC/MS.
  a. Seal the collection LoBind PCR plate containing eluted tryptic peptides with a new adhesive cover.
    ***Note:*** Peptide concentration can be measured before storage using either 1) Pierce Quantitative Colorimetric Peptide Assay, or 2) using a NanoDrop at A_205_ (Table 1).
  b. Store plate at −80°C.
    ***Note:*** At this stage, samples are ready for LC/MS analysis. This protocol is compatible with various LC/MS methods, provided they are designed for tryptic peptide analysis.

**Table 1:** Peptide concentration obtained by Nanodrop A_205_ and the number of proteins identified in 1 μg of each sample by LC-MS/MS

Sample	Nanodrop A205 (mg/ml)	Mass spectrometry (proteins identified in 1 μg)
Sample 1: Tear left, 1 min, Day 1	0.41	2594
Sample 2: Tear right, 1 min, Day 1	0.40	2832
Sample 3: Tear left, 1 min, Day 2	0.42	2924
Sample 4: Tear right, 1 min, Day 2	0.42	2965
Sample 5: Tear left, 1 min, Day 3	0.43	2939
Sample 6: Tear right, 1 min, Day 3	0.41	2902
Sample 7: Tear left, 5 min, Day 1	0.79	2861
Sample 8: Tear right, 5 min, Day 1	1.13	2975
Sample 9: Tear left, 5 min, Day 2	0.93	3104
Sample 10: Tear right, 5 min, Day 2	0.94	2963
Sample 11: Tear left, 5 min, Day 3	0.70	2673
Sample 12: Tear right, 5 min, Day 3	0.77	3024

## Expected outcomes

Protein concentration in human tear fluid can vary from person to person, with concentrations ranging from 6 to 11 g/L.^2^^,^^3^^,^^4^^,^^5^ Tear fluid can reflect physiological states of the body, such as glucose levels,^9^ as well as neurological and oncological conditions.^10^^,^^11^^,^^12^^,^^13^ Due to the ease of access, minimally invasive sampling methods, and relatively low cost, there is growing interest in utilizing tear fluid for biomarker studies. This protocol, using the standard Schirmer tear test (5 min absorption time) on average, yields ∼105 μg of tryptic peptides per eye/strip.

Here, tear samples were collected from both the right and left eyes of a single individual across three consecutive days (Day 1, Day 2, and Day 3) between 10:00 AM and 11:00 AM. At each time point, tear collection was performed using Schirmer strips for either **1 min** or **5 min**, to assess time-dependent variability. For each collection session, blank (unexposed) Schirmer strips were included as background controls to account for potential strip-related artifacts. Protein concentrations of all tear samples were quantified using a NanoDrop spectrophotometer (see Table 1). For downstream LC-MS/MS analysis, peptide eluates were dried using a centrifugal vacuum concentrator. The dried peptides were then resuspended, and 1 μg of the peptide mixture was injected into an EASY-nLC 1000 ultra-high-pressure liquid chromatography system (Thermo Fisher Scientific), coupled online to an Orbitrap Exploris 480 mass spectrometer.^14^^,^^15^

Normalized protein abundances (Spectronaut local normalization^16^) were loaded with R v4.30 [R Core Team, R: A Language and Environment for Statistical Computing. 2023.] and R package tidyverse v2.0.0. Tear sample protein normalization was checked with box plots, and tear sample separation from empty strip control samples was checked with Principal Component Analysis (PCA) scatter plots created by R function factorextra::fviz_pca_ind() v1.0.7. Correlation of abundances for proteins present in both left and right eyes, collected with the strip in situ for one-minute (days one to three) and for five minutes (days four to six), were tested with scatter plots and linear regression calculated with R function ggpmisc::state_ploy_eq() v0.5.6.

**Figure 5:**
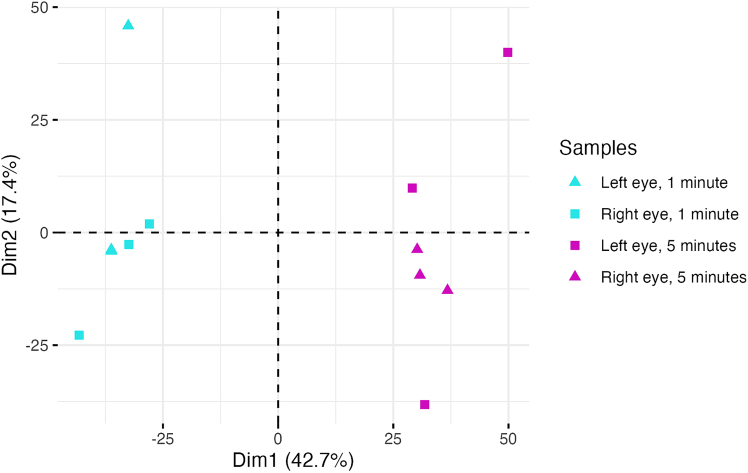
Principal component analysis (PCA) plot of tear samples Tear samples cluster distinctly based on collection duration, with 1-min samples (blue) separating from 5-min samples (purple). No significant clustering was observed based on eye laterality (triangle: left eye; square: right eye).

**Figure 6:**
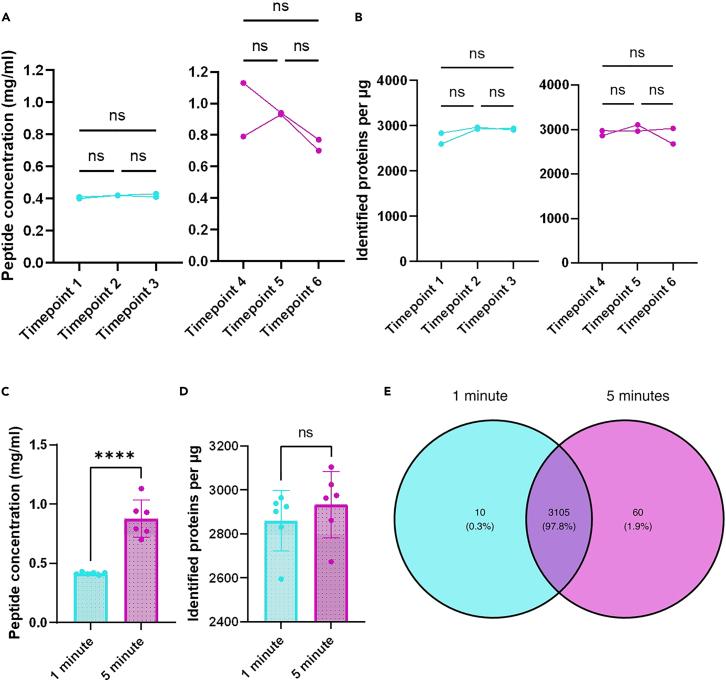
Comparative analysis of tear samples collected for 1 min (blue) and 5 min (purple) (A) Peptide concentrations (μg/μL) across timepoints 1–3 (1-min) and 4–6 (5-min). (B) Number of proteins identified in each sample. (C) Aggregate comparison of peptide concentrations between 1-min and 5-min collections. (D) Comparison of total proteins identified across collection durations. (E) Venn diagram showing 97.8% overlap in protein identifications between 1-min and 5-min samples. Statistics: Panels A and B: One-way ANOVA, p > 0.05; Panels C and D: unpaired *t* test, ∗∗∗∗ p < 0.0001). ns = not significant.

**Figure 7:**
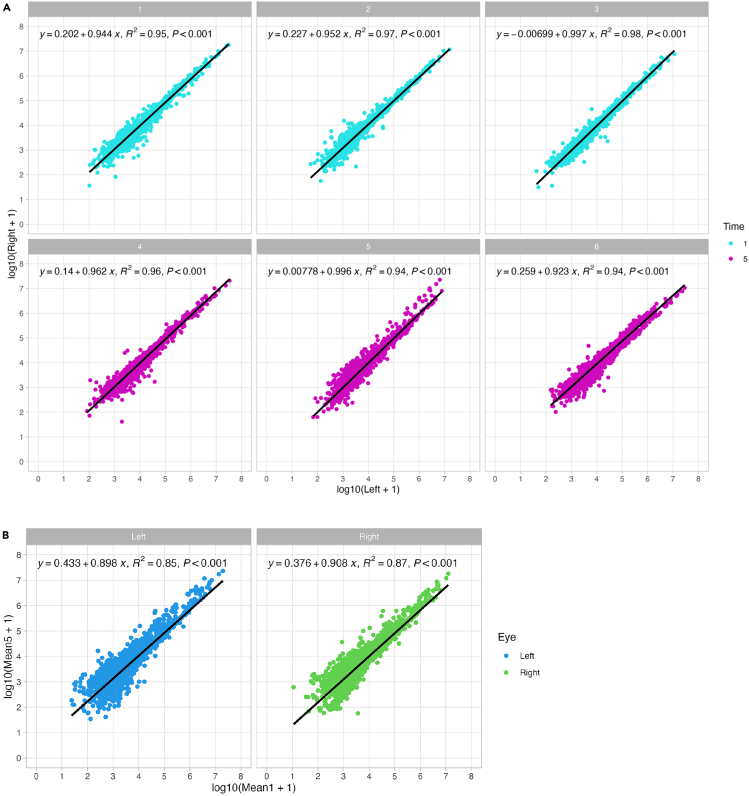
Correlation analysis of protein profiles in tear samples (A) Left vs. right eye protein profile correlations for each of the six collection timepoints (in blue, 1-min collections and purple, 5-min collections). (B) Protein profile correlations between 1-min and 5-min collection times for left (dark blue) and right (green) eyes. All comparisons demonstrate strong positive correlations (Pearson’s correlation coefficient, r). Each point represents an individual protein, with its abundance in the compared samples determining its position on the plot. Diagonal lines indicate significant correlation (r = 1).

This protocol enables consistent and minimally invasive tear fluid collection using Schirmer strips, yielding ∼105 μg of tryptic peptides per eye with a 5-min collection. LC-MS/MS analysis identified ∼2,896 proteins in tear samples, significantly more than in blank strips (∼186 proteins). Collection duration influenced peptide yield—5-min collections produced higher concentrations (876.67 μg/mL) than 1-min collections (415.00 μg/mL)—but did not affect the number or identity of proteins detected (Figures 5 and 6), which is consistent with previous reports.^17^^,^^18^^,^^19^ Strong correlations in protein abundance across all comparisons support the reproducibility of the method (Figure 7).

Although our results demonstrate that shorter collection times (e.g., 1 min) can yield comparable proteomic depth to longer durations, we recommend that a consistent sampling duration be maintained across all samples within a study. This ensures temporal uniformity and minimizes variability, particularly in comparative or longitudinal analyses. The consistency of tear composition across different eyes and time points demonstrates the effectiveness of the Schirmer strip collection and the subsequent protein digestion method used in tear proteomics studies. These results have significant implications for future research in translational tear proteomics. They suggest that simplified collection methods and high-throughput protein digestion protocols could be effective without a notable loss of proteomic information.

## Quantification and statistical analysis

The NanoPhotometer N60 (IMPLEN GmbH) is a general-purpose UV-Vis spectrophotometer, commonly used for nucleic acid and protein quantification. The peptide concentration was determined by measuring absorbance at 280 nm. Raw files were processed using the DirectDIA+ workflow with the library extension option enabled, using default settings in Spectronaut 18. Parametric unpaired t tests and One-way ANOVA were performed using GraphPad Prism 9.3.1, both assuming equal standard deviations.

## Limitations

This protocol relies on strict adherence to a standardized operating procedure (SOP) for sample collection, handling, and storage. Deviations—such as collecting samples at room temperature or directly on wet ice—may lead to protein degradation and reduced yield. Contamination risks include contact between the Schirmer strip and skin or the presence of makeup during collection, both of which can affect proteomic results. Consistency in collection methodology is essential; all samples must follow the same SOP to ensure reproducibility. While alternative tear collection methods (e.g., cotton threads, cellulose sponges) exist, this protocol does not evaluate their impact on protein recovery or downstream analysis. Additionally, the use of guanidine hydrochloride (GuaHCl) in this workflow may limit compatibility with multi-omics approaches, particularly those targeting nucleic acids, lipids, or metabolites.

## Troubleshooting

### Problem 1

**Interference of cosmetic products and makeup with downstream MS analysis**. The presence of makeup or other cosmetic products on Schirmer strip samples can compromise results.

### Potential solution

Establish and ensure adherence to this eligibility criterion for subjects donating tear fluid to avoid this issue (related to ‘Tear Sampling’ STEP 1). Educate all personnel involved in sample collection about the importance of makeup-free samples to ensure data integrity and provide clear instructions well in advance of sample collection.

### Problem 2

**Sample quality degradation due to inadequate freezing technique**. The failure to collect samples using dry ice may result in protein degradation, potentially leading to lower peptide yields and fewer proteins identified.

### Potential solution

Maintain strict dry ice protocol (related to ‘Tear Sampling’ STEP 1&2).

• Ensure all tear fluid samples are placed on dry ice immediately after collection.
• Maintain samples on dry ice during all handling and transportation steps.
• Triage samples from large-scale experiments by documenting the freezing time and conditions for each sample. Consider excluding samples with uncertain or delayed freezing history and, if needed, prioritize analysis of samples with verified immediate freezing.

The integrity of samples is crucial for reliable results. Establish and ensure adherence to standardized sample handling protocols to minimize variability in sample quality across experiments.

### Problem 3

**Issues with peptide yields.** When elution from the strip is not feasible, or using a needle is not an option, in-strip digestion can be performed.

### Potential solution

In-strip digestion (related to ‘Protein Digestion’ STEP 1). While it is possible to run this protocol without removing the strip after step 1c, that can lead to incomplete sample transfer to Sola plates due to liquid retention by the paper strip. However, if you are planning to analyze only half of your samples and keep the other half as backup, in-strip digestion could be a good solution to maximize peptide yields.

### Problem 4

**Suboptimal peptide digestion**. Incomplete peptide digestion can lead to a high number of missed cleavages, reducing the quality and of the mass spectrometry data.

### Potential solution

If mass spectrometry results show a high proportion of miss-cleaved peptides (related to ‘Protein Digestion’ STEP 4), the digestion time can be extended from the standard 1–1.5 h to an overnight incubation (∼12 h) period laboratory temperature (18°C–24°C).

Similarly, adjusting the enzyme-to-substrate ratio can significantly influence digestion efficiency. Slightly increasing the enzyme concentration will enhance digestion efficiency.

### Problem 5

**Achieving optimal sample acidification.** Sample acidification is crucial for downstream analysis, but the required volume of acidifying agent might need adjusting based on sample composition, size of Schirmer paper (strip vs disk) or collection method (depending on Schirmer strip branding).

### Potential solution

For the initial test, add 15–20 μL of acidifying agent (10% TFA) to one sample and measure pH by adding ∼2 μL of the acidified sample mixture on pH indicator strips. It may be that the sample still requires additional acidification (related to ‘Protein Digestion’ STEP 5). For subsequent adjustments, incrementally increase the volume by 2–5 μL per iteration. Monitor pH after each addition and adjust accordingly. Optimal acidification volume may slightly vary based on sample composition and the size of Schirmer paper. Calibrate and standardize for your specific experimental conditions (strips vs disks). Aim for the minimal TFA volume that achieves the target pH and document the final TFA volume for each sample type to streamline future experiments.

## Resource availability

### Lead contact

Further information and requests for resources and reagents should be directed to and will be fulfilled by the lead contact, Prof. Petra Hamerlik (petra.hamerlik@manchester.ac.uk).

### Technical contact

Technical questions on executing this protocol should be directed to and will be answered by the technical contact, Prof. Blagoy Blagoev (bab@bmb.sdu.dk).

### Materials availability

Data are available via ProteomeXchange with identifier PXD070688.

### Data and code availability

The in-house script is accessible through GitHub at https://github.com/Brain-Tumor-Biology/TearsProtocol.

## Acknowledgments

This work was funded by the Danish Cancer Institute (KBVU), the 10.13039/501100002203Brain Tumour Charity (GN-000612), 10.13039/501100000289CRUK Commercial Partnerships Project Development Fund, and 10.13039/501100000289Cancer Research UK (EDDPJT-May24/100005). The proteomics work was supported by the 10.13039/501100001732Danish National Research Foundation (DNRF141 to ATLAS) and the INTEGRA mass spectrometry research infrastructure funded by the 10.13039/501100009708Novo Nordisk Foundation (NNF20OC0061575). We thank Dr. Duncan Smith for their technical input in sample preparation and processing. The graphical abstract was created in BioRender. Hamerlik, P. (2025) https://BioRender.com/ucdvj7l.

## Author contributions

K.J.-E. and V.A. developed the in-strip digestion protocol and supervised further protocol optimization using SOLA plates. M.P.-P. and I.R.-P. collected samples for this study and conducted laboratory work. M.P.-P. analyzed the data. M.P.-P. and P.H. wrote the manuscript. V.A. and B.B. carried out the mass spectrometry analysis and protein search. M.R. performed the bioinformatic analysis. P.H. conceived the idea and designed the project plan.

## Declaration of interests

The authors declare no competing interests.
